# Adenotonsillectomy in Children with Obstructive Sleep Apnea Syndrome: Clinical and Functional Outcomes

**DOI:** 10.3390/jcm12185826

**Published:** 2023-09-07

**Authors:** Cristian Locci, Caterina Cenere, Giovanni Sotgiu, Mariangela Valentina Puci, Laura Saderi, Davide Rizzo, Francesco Bussu, Roberto Antonucci

**Affiliations:** 1 Pediatric Clinic, Department of Medicine, Surgery and Pharmacy, University of Sassari, 07100 Sassari, Italy; 2 Clinical Epidemiology and Medical Statistics Unit, Department of Medicine, Surgery and Pharmacy, University of Sassari, 07100 Sassari, Italy; 3 Otorhinolaryngology Operative Unit, Department of Medicine, Surgery and Pharmacy, University of Sassari, 07100 Sassari, Italy

**Keywords:** adenotonsillectomy, obstructive sleep apnea syndrome, quality of life, outcomes, children

## Abstract

Adenotonsillectomy (AT) is the first-line treatment for pediatric obstructive sleep apnea syndrome (OSAS). Relatively few studies have evaluated the clinical and functional outcomes of AT in children with OSAS, but these studies show that surgery improves behavior and quality of life (QOL). However, residual OSAS after AT is reported in severe cases. This study aimed to retrospectively evaluate the clinical and functional outcomes of AT in a cohort of children with OSAS. We consecutively enrolled children with OSAS who underwent AT and were admitted to our clinic from 1 July 2020 to 31 December 2022. For each participant, medical history and physical examinations were performed. Before and after surgery, all patients underwent a standard polygraphic evaluation, and caregivers completed the OSA-18 questionnaire. A total of 65 children with OSAS, aged 2–9 years, were included. After AT, 64 (98.4%) children showed a reduction in AHI, with median (IQR) values decreasing from 13.4/h (8.3–18.5/h) to 2.4/h (1.8–3.1/h) (*p*-value < 0.0001). Conversely, median (IQR) SpO_2_ nadir increased after surgery from 89% (84–92%) to 94% (93–95%) (*p*-value < 0.0001). Moreover, 27 children (18%) showed residual OSAS. The OSA-18 score decreased after AT from median (IQR) values of 84 (76–91) to values of 33 (26–44) (*p*-value < 0.0001). A positive significant correlation was found between OSA-18 post-operative scores and AHI post-operative scores (rho 0.31; *p*-value = 0.01). Our findings indicate that, in children with OSAS, AT is associated with significant improvements in behavior, QOL, and polygraphic parameters. However, long-term post-surgical follow-up to monitor for residual OSAS is highly recommended, especially in more severe cases.

## 1. Introduction

Obstructive sleep apnea syndrome (OSAS) is the most common sleep-related breathing disorder and is characterized by upper airway collapse that disrupts normal ventilation during sleep and normal sleep patterns [1]. OSAS is characterized by intermittent upper airway obstruction during sleep, which can lead to intermittent hypoxia, hypercapnia, increased respiratory effort with marked intrathoracic pressure swings, and repeated arousals causing sleep fragmentation [2]. The prevalence of OSAS among children is estimated to be 1–3% [3,4], and it is associated with several morbidities such as cardiovascular dysfunction [5,6], ventricular remodeling [7,8], endothelial dysfunction [9,10], neurobehavioral disturbances [11,12], enuresis [13], and somatic growth inhibition [14,15]. Moreover, OSAS in children affects parental psychological status, as it is associated with a significant level of anxiety depending on OSAS severity [16].

OSAS should be considered in the same way as inflammatory diseases: apneic events and intermittent hypoxia are associated with the overexpression of inflammatory markers and increased activation of the sympathetic system [17]. Immune dysregulation and recurrent infections in children may contribute to the development of adenotonsillar hypertrophy, which is a relevant risk factor for OSAS [18], by narrowing the upper airways resulting in snoring and obstructive sleep apnea.

Currently, overnight in-lab polysomnography (PSG) is the gold standard for the diagnosis of OSAS. However, it is expensive and often not available in all centers. Nevertheless, home cardiorespiratory polygraphy (HRP) has proven to be a potentially valuable and reliable approach [19].

Adenotonsillectomy (AT) is the first-line treatment for moderate to severe OSAS [20,21], and it remains one of the most common surgical procedures performed in children. The outcome of AT for OSAS can be evaluated based on objective evidence provided by pre-operative and post-operative PSG. Several studies have shown that both the behavior and the quality of life (QOL) of children with OSAS can improve after AT in most cases [22,23,24,25,26,27,28]. However, in recent years, the persistence of abnormal PSG findings suggestive of residual OSAS is reported in approximately 20–40% of patients, particularly in severe cases [15,29,30,31,32,33,34,35]. In the literature, there are relatively few studies investigating outcomes of AT in pediatric OSAS [15]. Some studies showed an improvement in QOL after AT but did not evaluate the correlation between PSG parameters and QOL score [36]. In other studies, PSG data were correlated with pre- and post-operative findings obtained using the Obstructive Sleep Apnea 18-Item Quality-of-Life Questionnaire (OSA-18) instrument [31,37,38].

This study aimed to retrospectively evaluate functional and clinical outcomes of AT in a cohort of children with OSAS, using standard overnight in-hospital polygraphy (PG) and the OSA-18 questionnaire before and after surgery.

## 2. Materials and Methods

From 1 July 2020 to 31 December 2022, we consecutively enrolled Caucasian children affected by OSAS, who were referred to the pediatric pulmonology service at the Pediatric Clinic of the University of Sassari, Italy, and underwent AT based on preoperative OSA-18 and PG findings. Exclusion criteria included genetic or craniofacial syndromes, neuromuscular diseases, and a BMI z-score of ≥3. Moreover, children for whom caregivers failed to complete the OSA-18 questionnaire were excluded. For each enrolled child, complete medical history and physical examination were performed by a single trained investigator. Demographic and clinical data including age, gender, BMI z-score, tonsil size grading, palate conformation (Friedman Palate Position), nasal obstruction, mouth breathing, and presence of inhalant allergen sensitization were obtained. The skin prick test (SPT) and total IgE were used to detect the sensitization to allergens.

Age- and sex-specific BMI percentiles based on Italian cross-sectional growth charts [39] were obtained. According to these reference growth charts, subjects with a BMI between the 85th and 94th percentiles were classified as overweight, while those with BMI ≥ the 95th percentile (BMI z-score greater than 1.65) were categorized as obese [40].

For each participant, a caregiver was asked to complete the OSA-18 questionnaire [41,42] just before PG, both before and after AT.

This questionnaire includes 18 items in 5 domains: sleep disturbance, physical suffering, emotional distress, daytime problems, and caregiver concerns. The questionnaire provides a score ranging from 18 to 126, with higher values indicating worse QOL.

All enrolled children underwent standard overnight in-hospital PG (SOMNOscreen™ Plus, SOMNOmedics GmbH, Randersacker, Germany) before and after AT, with the assessment of the following parameters: oro–nasal airflow, snoring, thoracic and abdominal movements (inductance plethysmography), pulse oximetry, and body position. A post-operative polygraphic examination was carried out between 180 and 900 days from the date of the intervention.

The results of PG were evaluated according to the American Academy of Pediatrics and American Academy of Sleep Medicine guidelines for pediatric OSAS [21,43]. AHI was calculated as the number of apnea and hypopnea episodes per hour of sleep (events/h). For this study, the diagnosis of OSAS was defined as AHI ≥ 2 events/h of total sleep time (TST). The severity of OSAS was classified as follows: mild OSAS (AHI < 5/h); moderate OSAS (5/h ≤ AHI < 10/h); severe OSAS (AHI ≥ 10/h). A post-operative value of AHI ≥ 3/h was considered indicative of residual OSAS. The SpO_2_ nadir was defined as the lowest value of oxygen saturation detected in TST [21,43].

The study population was divided into groups, based on the age and OSAS severity, to evaluate whether they influenced the considered variables.

The majority of study participants underwent AT for moderate to severe OSAS. However, regardless of OSAS severity, AT was also carried out in patients with recurrent tonsillitis and comorbidities such as dysphagia, failure to thrive, high-grade tonsillar hypertrophy, general poor health, enuresis, asthma, and behavioral problems, according to the guidelines of the American Academy of Otolaryngology–Head and Neck Surgery [22].

### Statistical Analysis

Sample characteristics were described using mean and standard deviations (SDs) or medians and 25°–75° percentiles (IQR) in the case of quantitative variables, whereas absolute and relative (percentages) frequencies were used for qualitative variables. Subgroup differences among quantitative variables were evaluated using Student’s t test or Mann–Whitney tests. Pearson Chi-square or Fischer exact tests were used to assess differences in qualitative variables. The comparison of variables among OSAS severity levels (i.e., 3 groups) was carried out using one-way ANOVA or its equivalent nonparametric version (i.e., Kruskall–Wallis test). In the case of significant ANOVA, a post hoc comparison was performed. Crude odds ratios (ORs) with 95% confidence intervals were calculated to evaluate the association between variables and OSAS. Spearman’s correlation was used to evaluate the relationships between OSA-18 and AHI, before and after AT.

Two-tailed *p*-value less than 0.05 was considered statistically significant, statistical analyses were carried out using STATA version 17 (StataCorp. 2021. Stata Statistical Software: Release 17. StataCorp LLC., College Station, TX, USA).

## 3. Results

In the study period, a total of 65 children aged 2–9 years, with a prevalence of males (66%), were enrolled. The median (IQR) age was 4 (4–6) years, and the median (IQR) BMI z-score was −0.27 (−1.18; 0.32) (Table 1). Rhinitis was found in less than a quarter (24%) of the subjects evaluated, with a relatively low percentage (13%) of allergen sensitization diagnosed through immediate-reading SPT. The concomitant presence of asthma was detected in only 5% of patients. Over half (51%) of study participants had high-grade adenotonsillar hypertrophy, with predominantly oral breathing. Night snoring was present in 61% of patients, and nasal obstruction in 55%. 

The severity of OSAS, classified based on PG, was distributed as follows: 69.2% of patients had severe OSAS, 24.6% moderate OSAS, and 6.2% mild OSAS. Regarding polygraphic parameters, the median (IQR) pre-operative AHI was 13.4/h (8.3–18.5), while the median pre-operative SpO_2_ nadir was 89% (84–92%) in room air.

**Figure 1:**
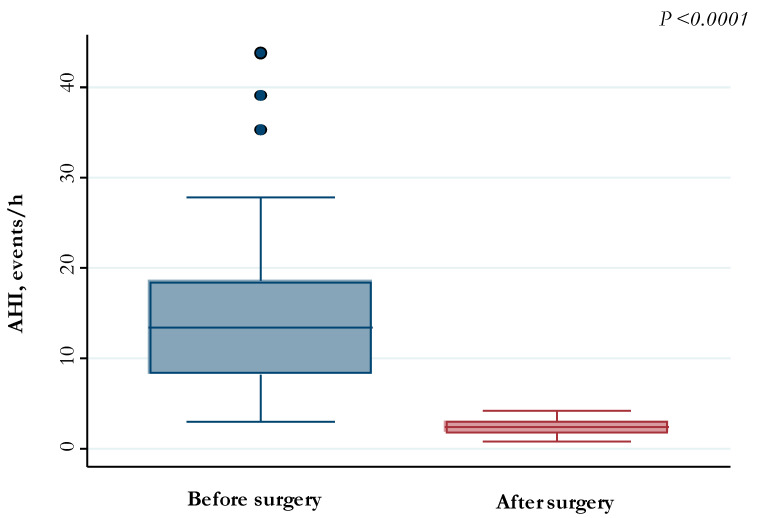
AHI values measured before and after adenotonsillectomy.

**Figure 2:**
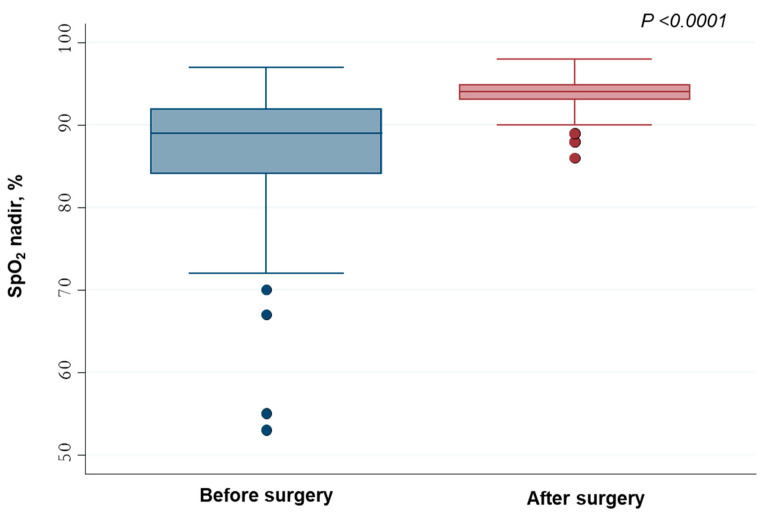
SpO_2_ nadir values measured before and after adenotonsillectomy.

After AT, 64 (98.4%) children showed a reduction in AHI, with median (IQR) values decreasing from 13.4/h (8.3–18.5/h) to 2.4/h (1.8–3.1/h) (*p*-value < 0.0001) (Figure 1). Conversely, median (IQR) SpO_2_ nadir increased after surgery from 89% (84–92%) to 94% (93–95%) (*p*-value < 0.0001) (Figure 2). 

Considering an AHI cut-off value of less than 3 events/h of TST, we found that breathing patterns during sleep were not fully normalized in 27 (18%) patients, who showed residual OSAS.

**Figure 3:**
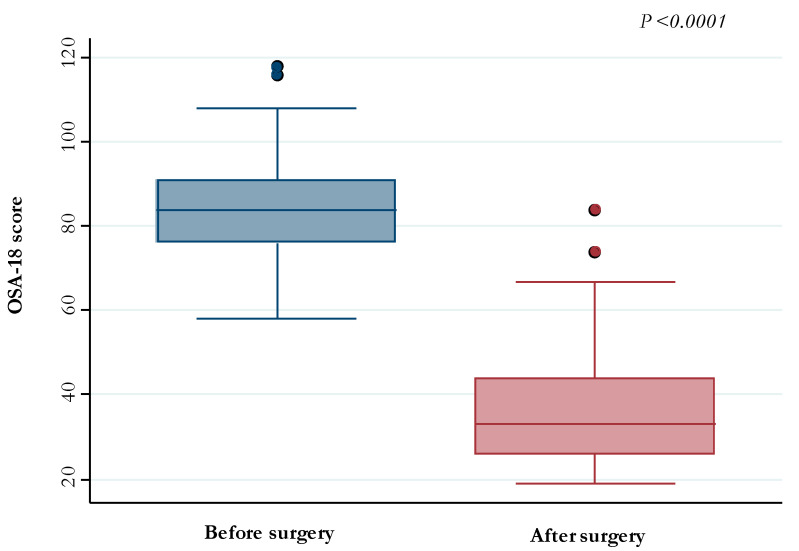
OSA-18 scores detected before and after adenotonsillectomy.

Finally, the OSA-18 score decreased after AT from median (IQR) values of 84 (76–91) to values of 33 (26–44) (*p*-value <0.0001) (Figure 3). 

No significant difference in pre-operative and post-operative values of variables was found when comparing the age group <5 years to that ≥5 years (Table 2). 

Regarding the severity of OSAS, we found a significant difference in tonsil size score (88.9%, *p*-value = 0.005) and Friedman palate position (51%, *p*-value = 0.01) between the three severity groups, with higher values (III–IV) associated with the severe OSAS group. Moreover, oral breathing (93.3%, *p*-value < 0.0001), nasal airway patency (91.1%, *p*-value = 0.002), and snoring (100.0%, *p*-value = 0.001), were significantly higher in this group (Table 3). The median (IQR) pre-operative SpO_2_ nadir was significantly lower in patients with severe OSAS (86%; 79–90%) than in those with mild (93%; 89–95%) and moderate (92.5%; 91–94%) OSAS (*p*-value = 0.0001). 

Furthermore, residual OSAS (post-operative AHI ≥ 3/h) was found to be more frequent in patients with severe OSAS when compared to those with mild or moderate OSAS, although not significantly. There were no significant differences for any of the other variables between the three OSAS severity groups.

The pre-operative AHI scores had a nonsignificant correlation with the pre-operative OSA-18 scores (rho 0.19; *p*-value = 0.13), whereas a positive significant correlation was found between OSA-18 post-operative scores and AHI post-operative scores (rho 0.31; *p*-value = 0.01) (Table 4). 

A univariate logistic analysis was carried out to assess the relationships between demographic and clinical variables, and residual OSAS following AT. Male sex and Friedman palate position III–IV (but not tonsil size grading III–IV) were significantly associated with residual OSAS. There were no significant associations between the remaining variables and residual OSAS (Table 5). 

## 4. Discussion

Pre-operative PSG is an essential diagnostic tool for identifying children with severe OSAS, in whom AT is indicated. However, when PSG is not readily available, some authors have proposed a formula to assess the severity of OSAS using the Mallampati score and tonsil size grading [44]. Moreover, a comparison of clinical data with PSG revealed that Mallampati score and tonsil size were significantly associated with AHI [45].

In this study, more than two-thirds of the children who underwent surgery were affected by severe OSAS. This finding appears to be substantially in line with published data [31,37,38]. Surgical treatment of children with OSAS is the first choice according to the American Academy of Pediatrics, particularly in OSAS with AHI > 10/h [21,46]. The evidence on the efficacy of AT in children with OSAS derives from observational studies [47], given the difficulty of performing randomized controlled trials and ethical reasons. The available data consistently indicate that the intervention is effective in the treatment of children with OSAS associated with adenotonsillar hypertrophy, without significant comorbidities. A systematic review including 14 case series [27] indicated that AT led to a normalization of polysomnographic parameters (AHI and SpO_2_ nadir).

In our study population, AT resulted in a significant reduction in AHI values (Figure 1), similarly to other studies [15,31,38]. Moreover, the values of SpO_2_ nadir after AT were found to be significantly higher than pre-operative values (Figure 2).

Some meta-analyses indicated that AT is not completely effective in the treatment of OSAS in children [27,28,48], and residual OSAS was reported in older children and obese children [38].

A recent systematic review found a significant reduction in post-operative AHI values in children undergoing AT for severe OSAS. Moreover, all the studies examined by the investigators documented a variable proportion of residual OSAS following AT [49]. In the meta-analysis by Brietzke [27], the percentage of successfully treated children was 83%. Our study found residual OSAS (post-operative AHI ≥ 3/h) in 27 (18%) children, more frequently in patients with severe OSAS compared to those with mild or moderate OSAS, although statistical significance was not reached.

Previous studies demonstrated that there is a relationship between the severity of OSAS, as determined by pre-operative AHI, and subsequent surgical response [31,35,50].

Other prospective studies showed that OSAS leads to the deterioration of cognitive and behavioral skills and QOL of children; conversely, AT is effective in resolving sleep disturbances with the normalization of AHI [25,51,52,53], improving academic performance, cognitive/behavioral skills [54,55,56], and QOL [25,26,54,57,58], with results being maintained over time [59,60].

There are limited data on the correlation between PSG parameters and QOL in OSAS children. Several studies assessed the relationship between the severity of OSAS (measured as AHI) and QOL in children [25,26,59]. In particular, a poor correlation between the severity of OSAS and OSA-18 score has been documented [61,62,63]. In the present study, a significant reduction in the OSA-18 score was observed following AT (Figure 3). Previous studies found that OSA-18 was not correlated with AHI in pediatric patients, appearing instead to be a tool to evaluate the outcome of AT [64,65,66]. Studies using the OSA-18 as a tool to detect OSAS have provided contrasting results. The questionnaire is probably more useful in combination with other diagnostic tools and cannot replace the PSG [67].

Regarding the correlation between PSG scores and OSA-18, in partial disagreement with the results of previous studies [37,38], our findings show that pre-operative AHI values have a nonsignificant correlation with pre-operative OSA-18 scores, whereas a significant positive correlation between post-operative OSA-18 score and post-operative AHI values was found.

To evaluate the influence of age on the variables investigated in OSAS patients, we compared children aged <5 years to those aged ≥5 years, but no significant difference was found between the two groups. Of note, the majority (64%) of study participants were aged less than 5 years, which is consistent with the peak incidence of OSAS reported in the literature [21]. The absence of significant differences between the two age groups might be the result of a relatively small number of study participants.

In line with the study by Overland et al. [37], we found a significant difference in tonsil size score, Friedman palate position, oral breathing, nasal airway patency, and snoring, between the three groups with different OSAS severity, with higher values being associated with severe OSAS. Moreover, children with severe OSAS had a significantly lower pre-operative SpO_2_ nadir than those with mild or moderate OSAS. Some authors suggested that the degree of hypoxemia (SpO_2_ nadir) before AT, rather than AHI, may be a better predictor for residual OSAS [68]. Regarding other factors such as age, male sex, allergy, and asthma, conflicting data have been reported [38,68,69,70]. The study by Ye et al. [38] showed that residual OSAS was associated with obesity, severe OSAS before surgery, greater tonsillar hypertrophy, allergen sensitization, and asthma. In our study, a univariate logistic analysis showed that only male sex and Friedman palate position III–IV (but not tonsil size grading III–IV) were significantly associated with residual OSAS (AHI ≥ 3) after AT; none of the other analyzed variables was associated with a significant increase in the risk of residual OSAS (Table 5).

The retrospective observational design, relatively small sample size, and use of a single center are the main limitations of this study.

## 5. Conclusions

In conclusion, our findings provide evidence that AT is associated with significant improvements in behavior, QOL, and PG parameters, in children with OSAS, particularly in the most severe cases. However, special attention should be paid to the risk of residual OSAS after surgery; for this reason, long-term post-surgical clinical and polysomnographic follow-up is highly recommended. In the future, more extensive prospective studies will need to shed further light on the unresolved issue of pathogenesis of residual OSAS in order to optimize AT effectiveness.

## Figures and Tables

**Table 1:** Variables analyzed in the OSAS children included in the study.

Variables		Total Cohort (*n* = 65)
Males, *n* (%)		43 (66.2)
Median (IQR) age at diagnosis, years		4 (4–6)
Median (IQR) weight, kg		17 (15–20)
Mean (SD) height, cm		107 (11.7)
Median (IQR) BMI z-score		−0.27 (−1.18; 0.32)
Median (IQR) BMI, kg/m^2^		15.4 (14.2–16.6)
Median (IQR) pre-operative AHI, events/h		13.4 (8.3–18.5)
Severity levels of OSAS	Mild OSAS (AHI < 5)	4 (6.2)
	Moderate OSAS (AHI 5–9.9)	16 (24.6)
	Severe OSAS (AHI ≥ 10)	45 (69.2)
Mean (SD) post-operative AHI, events/h		2.4 (0.9)
Post-operative AHI ≥ 3, *n* (%)		18 (27.7)
Tonsil size grading III–IV, *n* (%)		51 (78.5)
Friedman palate position III–IV, *n* (%)		26 (40.0)
Median (IQR) pre-operative SpO_2_ nadir, %		89 (84–92)
Median (IQR) post-operative SpO_2_ nadir, %		94 (93–95)
Oral breathing, *n* (%)		53 (81.5)
Nasal airway patency, *n* (%)		55 (84.6)
Snoring, *n* (%)		61 (93.9)
Allergen sensitization, *n* (%)		13 (20.0)
Asthma, *n* (%)		5 (7.7)
Rhinitis, *n* (%)		24 (36.9)
Median (IQR) pre-operative OSA-18		84 (76–91)
Median (IQR) post-operative OSA-18		33 (26–44)

BMI: Body Mass Index. AHI: Apnea–Hypopnea Index. IQR: interquartile range.

**Table 2:** Clinical and epidemiological variables in two age groups of OSAS children.

Variables		Age < 5 Years (*n* = 42)	Age ≥ 5 Years (*n* = 23)	*p*-Value
Males, *n* (%)		28 (66.7)	15 (65.2)	0.91
Median (IQR) BMI z-score		−0.48 (−1.32; 0.24)	−0.06 (−1.13; 1.12)	0.19
Median (IQR) BMI, kg/m^2^		15.2 (14.1–16.2)	16.0 (14.4–19.8)	0.08
Median (IQR) pre-operative AHI, events/h		13.6 (8.8–18.0)	12.1 (8.1–19.6)	0.58
Severity levels of OSAS, *n* (%)	Mild OSAS (AHI < 5)	2 (4.8)	2 (8.7)	0.75
	Moderate OSAS (AHI 5–9.9)	10 (23.8)	6 (29.1)	
	Severe OSAS (AHI ≥ 10)	30 (71.4)	15 (65.2)	
Median (IQR) post-operative AHI, events/h		2.4 (1.6–2.9)	2.6 (1.8–3.3)	0.49
Post-operative AHI ≥ 3, *n* (%)		10 (23.8)	8 (34.8)	0.34
Tonsil size grading III–IV, *n* (%)		34 (81.0)	17 (73.9)	0.51
Friedman palate position III–IV, *n* (%)		16 (38.1)	10 (43.5)	0.67
Median (IQR) pre-operative SpO_2_ nadir, %		89 (86–92)	89 (81–92)	0.91
Median (IQR) post-operative SpO_2_ nadir, %		94 (93–95)	94 (93–95)	0.83
Oral breathing, *n* (%)		37 (88.1)	16 (69.6)	0.07
Nasal airway patency, *n* (%)		37 (88.1)	18 (79.3)	0.31
Snoring, *n* (%)		39 (92.9)	22 (95.7)	1.00
Allergen sensitization, *n* (%)		6 (14.3)	7 (30.4)	0.12
Asthma, *n* (%)		2 (4.8)	3 (13.0)	0.34
Rhinitis, *n* (%)		13 (31.0)	11 (47.8)	0.18
Median (IQR) pre-operative OSA-18		85.5 (79–91)	81 (69–93)	0.15
Median (IQR) post-operative OSA-18		35 (27–45)	31 (24–36)	0.19

BMI: Body Mass Index. AHI: Apnea–Hypopnea Index. IQR: interquartile range.

**Table 3:** Clinical and epidemiological variables in three classes of children with different OSAS severity.

Variables	Mild OSAS AHI < 5 (*n* = 4)	Moderate OSAS AHI 5–9.9 (*n* = 16)	Severe OSAS AHI ≥ 10 (*n* = 45)	*p*-Value
Males, *n* (%)	2 (50.0)	11 (68.8)	30 (66.7)	0.82
Median (IQR) age at diagnosis, years	5 (3–6)	4 (3.5–6.5)	4 (4–5)	0.95
Median (IQR) weight, kg	17.8 (14.5–19.3)	17.5 (15–25)	17 (15.0–19.7)	0.74
Median (IQR) height, cm	108 (93.5–116.0)	111 (98.0–117.5)	105 (101–113)	0.89
Median (IQR) BMI z-score	−0.10 (−0.69; 0.53)	−0.08 (−1.01; 0.80)	−0.29 (−1.32; 0.24)	0.54
Median (IQR) BMI, kg/m^2^	16.0 (14.9–17.2)	15.8 (14.4–18.2)	15.4 (14.1–16.2)	0.34
Median (IQR) post-operative AHI, events/h	1.9 (1.2–3.0)	2.3 (1.7–2.7)	2.5 (1.9–3.2)	0.51
Postoperative AHI ≥ 3, *n* (%)	1 (25.0)	3 (18.8)	14 (31.1)	0.79
Tonsil size grading III–IV, *n* (%)	3 (75.0)	8 (50.0)	40 (88.9)	0.005
Friedman palate position III–IV, *n* (%)	1 (25.0)	2 (12.5)	23 (51.1)	0.01 **^(1)^**
Median (IQR) pre-operative SpO_2_ nadir, %	93 (89–95)	92.5 (91–94)	86 (79–90)	0.0001 **^(2)^**
Median (IQR) post-operative SpO_2_ nadir, %	95 (94.5–95.5)	94.5 (93–95.5)	94 (93–95)	0.38 **^(3)^**
Oral breathing, *n* (%)	1 (25.0)	10 (62.5)	42 (93.3)	<0.0001 **^(4)^**
Nasal airway patency, *n* (%)	1 (25.0)	13 (81.3)	41 (91.1)	0.002 **^(5)^**
Snoring, *n* (%)	2 (50.0)	14 (87.5)	45 (100.0)	0.001 **^(6)^**
Allergen sensitization, *n* (%)	1 (25.0)	4 (25.0)	8 (17.8)	0.67
Asthma, *n* (%)	0 (0.0)	2 (12.5)	3 (6.7)	0.71
Rhinitis, *n* (%)	1 (25.0)	7 (43.8)	16 (35.6)	0.76
Median (IQR) pre-operative OSA-18	84.5 (70.5–88.5)	78 (73–93)	84 (79–91)	0.69
Median (IQR) post-operative OSA-18	50 (29–68)	37 (24.5–48.5)	31 (26–41)	0.33

**^(1)^** Moderate OSAS vs. Severe OSAS *p* = 0.007. **^(2)^** Mild OSAS vs. Severe OSAS *p* = 0.03; Moderate OSAS vs. Severe OSAS *p* < 0.0001. ^(**3)**^ Moderate OSAS vs. Severe OSAS *p* < 0.0001. **^(4)^** Mild OSAS vs. Severe OSAS *p* = 0.0001; Moderate OSAS vs. Severe OSAS *p* = 0.003. **^(5)^** Mild OSAS vs. Moderate OSAS *p* = 0.03; Mild OSAS vs. Severe OSAS *p* = 0.0003. **^(6)^** Mild OSAS vs. Severe OSAS *p* < 0.0001; Moderate OSAS vs. Severe OSAS *p* = 0.02.

**Table 4:** Spearman’s correlation coefficients between OSA-18 and AHI, before and after adenotonsillectomy.

Variables	Pre-Operative AHI	Post-Operative AHI
Pre-operative OSA-18, rho (*p*-value)	0.19 (0.13)	-
Post-operative OSA-18, rho (*p*-value)	-	0.31 (0.01)

**Table 5:** Univariate analysis to assess the relationship between demographic and clinical variables, and residual OSAS (post-operative AHI ≥ 3).

Variables		Univariate Analysis	
		OR (95% CI)	*p*-Value
Males		13.7 (1.69–111.8)	0.01
Age at diagnosis, years		1.09 (0.77–1.53)	0.63
Weight, kg		1.04 (0.96–1.13)	0.36
Height, cm		1.02 (0.97–1.07)	0.47
BMI z-score		1.42 (0.88–2.29)	0.16
BMI, kg/m^2^		1.17 (091–1.50)	0.21
Pre-operative AHI, events/h		1.06 (0.99–1.13)	0.09
Severity levels of OSAS	Mild OSAS (AHI < 5)	Ref.	Ref.
	Moderate OSAS (AHI 5–9.9)	0.69 (0.05–9.21)	0.78
	Severe OSAS (AHI ≥ 10)	1.36 (0.13–14.2)	0.80
Tonsil size grading III–IV		0.95 (0.26–3.51)	0.94
Friedman palate position III–IV		3.35 (1.08–10.4)	0.04
Pre-operative SpO_2_ nadir, %		0.97 (0.91–1.03)	0.24
Oral breathing		0.72 (0.19–2.76)	0.63
Nasal airway patency		1.64 (0.31–8.59)	0.56
Snoring		1.16 (0.11–11.9)	0.90
Allergen sensitization		2.86 (0.81–10.1)	0.10
Asthma		0.63 (0.07–6.07)	0.69
Rhinitis		1.12 (0.37–3.44)	0.84

BMI: Body Mass Index. AHI: Apnea–Hypopnea Index.

## Data Availability

The datasets generated and/or analyzed during the current study are available from the corresponding author upon reasonable request.

## Abbreviations

OSAS	obstructive sleep apnea syndrome
AHI	Apnea–Hypopnea Index
PG	polygraphy
PSG	polysomnography
HRP	home cardiorespiratory polygraphy
BMI	Body Mass Index
TST	total sleep time
IQR	interquartile range
